# Chrononutrition, Body Composition, and Resting Metabolic Rate Among College Students: A Cross-Sectional Study

**DOI:** 10.3390/nu18081214

**Published:** 2026-04-11

**Authors:** Kun Xu, Shuo Yan, Yuqin Ji, Yihan Meng, Hongjuan Li

**Affiliations:** Key Laboratory of the Ministry of Education of Exercise and Physical Fitness, School of Sport Science, Beijing Sport University, Beijing 100084, China; 13919371750@163.com (K.X.);

**Keywords:** chrononutrition, body composition, resting metabolic rate, sports-majoring college students, Chinese young adults

## Abstract

Background: Chrononutrition is essential for metabolic health, but relevant evidence in Chinese sports-majoring college students is still insufficient. This study aimed to identify chrononutrition patterns and their associations with body composition and resting metabolic rate (RMR) in college students from a sports university. Methods: A cross-sectional study was conducted among 174 college students from Beijing Sport University (131 sports-majoring and 43 non-sports-majoring). Chrononutrition was measured by the validated Chinese version of the Chrononutrition Profile Questionnaire (CP-Q), body composition by dual-energy X-ray absorptiometry, and RMR by indirect calorimetry. Sample sizes varied across analyses according to data availability, and 133 participants provided valid data for both body composition and resting metabolic rate (RMR) assessments. Results: Frequent night eating was positively correlated with BMI (r = 0.27, *p* = 0.001), and regular breakfast consumption was related to higher muscle mass percentage (β = 0.23, *p* < 0.01, sr^2^ = 0.05). Compared with non-sports-majoring students, sports-majoring students had longer weekday eating windows (11.2 ± 2.8 h vs. 8.5 ± 2.5 h, *p* < 0.001) and a higher dinner energy proportion (37.2 ± 6.9% vs. 30.5 ± 6.5%, *p* < 0.001). Males had later meal times and longer eating windows than females (breakfast: 7:58 vs. 7:46; dinner: 18:55 vs. 18:41; eating window: 11.5 h vs. 10.9 h; all *p* < 0.05). Conclusions: Chrononutrition was more closely associated with body composition than with absolute RMR in this predominantly sports-majoring sample of Chinese college students. Regular breakfast and reduced night eating are potential intervention targets for future chrononutrition guidance. However, the findings should be generalized to the broader college student population with caution.

## 1. Introduction

Chrononutrition is an emerging area of nutritional science that focuses on not only what and how much people eat, but also on when, how regularly, and across what duration eating occurs within the 24 h day [1,2]. Recent evidence has identified that meal timing, eating regularity, eating duration, and alignment between eating and sleep–wake rhythms are core dimensions of chrononutrition that may contribute to cardiometabolic health and obesity prevention [3,4], suggesting that the temporal organization of eating is relevant to weight regulation and metabolic health [5,6]. Thus, chrononutrition provides a useful framework for understanding how the temporal organization of eating may affect health beyond conventional nutrient-based approaches.

The biological rationale for chrononutrition lies in the close interaction between eating behavior and the circadian timing system. Circadian rhythms regulate a wide range of physiological processes, including endocrine secretion, substrate utilization, and energy metabolism, whereas meal timing functions as an important behavioral cue for peripheral metabolic rhythms [7,8]. Consequently, delayed, irregular, or poorly aligned eating schedules may disrupt the coordination between circadian rhythms and metabolic regulation, thereby contributing to impaired metabolic homeostasis and increased risks of obesity [9] and cardiovascular diseases [10].

Recent evidence has further emphasized that the temporal characteristics of eating are closely intertwined with sleep timing and sleep health [11], suggesting that chrononutrition should be understood within a broader behavioral circadian context rather than as an isolated dietary factor.

Growing evidence suggests that chrononutrition is relevant to body composition regulation. Observational and intervention studies indicate that regular meals, breakfast consumption, and appropriately timed weekday eating windows may be associated with more favorable adiposity-related outcomes [12,13,14,15], while observational work in female university students has shown that breakfast skipping is associated with higher body fat and lower fat-free mass even in those with normal BMI [16]. Collectively, these findings support the possibility that temporal eating behaviors are related not only to adiposity, but also to lean-mass-related phenotypes.

Resting metabolic rate (RMR) is another important component of metabolic health because it constitutes a major proportion of total daily energy expenditure and is strongly influenced by body composition [17]. Recent work on human energy balance suggests that meal timing may also affect metabolic regulation, and that late eating may be linked to lower metabolic efficiency and biological changes favoring fat storage [18,19]. Although the evidence directly linking chrononutrition with absolute RMR remains less established than that relating chrononutrition to obesity and body composition, the available literature provides a plausible theoretical basis for investigating this association.

College students are a population of particular interest in chrononutrition research because they commonly experience irregular schedules, delayed bedtimes and wake times, breakfast skipping, late eating, and unstable daily routines. These behavioral characteristics may place them at increased risk of circadian eating disruption and subsequent metabolic imbalance. Supporting this concern, recent research among Japanese college students found that irregular mealtimes were associated with social and eating jet lag [16,20]. However, compared with the growing international literature, evidence regarding chrononutrition patterns and their associations with body composition [21] and RMR in Chinese college students remains limited. Notably, sports-majoring students represent a highly active population with distinct energy requirements, muscle-driven body composition, and post-exercise nutrient needs—yet their chrononutrition profiles remain largely uncharacterized. Conventional findings from general college students cannot be directly extrapolated to this group due to fundamental differences in metabolism, activity levels, and daily eating routines. Therefore, this study specifically focuses on predominantly sports-majoring college students at Beijing Sport University, aiming to explore the chrononutrition patterns and their metabolic associations in this unique population, and provide targeted references for subsequent research in the general college student group.

Therefore, the present study aimed to describe chrononutrition patterns among predominantly sports-majoring Chinese college students and to examine whether key chrononutrition indicators were associated with body composition and resting metabolic rate (RMR), with attention to differences by sex and academic major. We hypothesized that: (1) Chrononutrition behaviors would be more strongly associated with body composition than with absolute RMR; (2) Sports-majoring students would exhibit different chrononutrition patterns, including longer weekday eating windows and a higher dinner energy proportion, than non-sports-majoring students; (3) Sex differences exist in chrononutrition patterns, with males showing later meal times and longer weekday eating windows. This study may help extend the current chrononutrition literature to a Chinese young-adult population with high physical activity levels and provide evidence for future nutrition and lifestyle interventions in college students.

## 2. Materials and Methods

### 2.1. Study Design and Participants

A cross-sectional survey was conducted from April 2025 to July 2025, with convenience sampling used to recruit full-time undergraduate and postgraduate students from Beijing Sport University. A total of 174 students were enrolled, including 64 males (36.8%) and 110 females (63.2%). Of these, 131 were sports-majoring students from physical education, competitive sports, and sports training programs. According to self-reported physical activity data collected using the International Physical Activity Questionnaire-Short Form (IPAQ-SF), this group generally reported higher habitual physical activity levels. The remaining 43 were non-sports-majoring students from sports management and sports science programs, who generally reported lower activity levels. To reduce selection bias in convenience sampling, recruitment was balanced across grades, majors, and sex as far as feasible. No significant differences in age, gender or BMI were observed between sports-majoring and non-sports-majoring participants in the enrolled sample. Although the eligibility criteria included students aged 18–29 years, the final enrolled sample ranged from 18 to 24 years.

Inclusion criteria: (1) Full-time undergraduate or postgraduate students aged 18–29 years; (2) Voluntarily participate in the study and sign the informed consent form; (3) No severe metabolic diseases such as heart, liver or kidney diseases, no endocrine diseases (e.g., abnormal thyroid function), and no mental disorders; (4) No intake of drugs affecting metabolism and body composition (e.g., hormonal drugs, hypoglycemic drugs, anabolic steroids) and no participation in deliberate weight loss or muscle gain training (≥3 times/week, ≥60 min/time) in the past 3 months; (5) Able to cooperate with completing questionnaires, body composition tests and RMR tests; (6) No long-term smoking (≥1 cigarette/day for more than 6 months) or excessive drinking (≥50 g of alcohol/day for more than 3 months).

Exclusion criteria: (1) Subjects who do not meet the above inclusion criteria; (2) Questionnaires with incomplete filling (missing rate > 30%) or logical confusion that cannot be used for data analysis; (3) Subjects with invalid test data due to non-cooperation during the testing process (e.g., movement during DXA scanning, irregular breathing during RMR measurement); (4) Subjects with extreme values of body composition or RMR (±3 SD from the mean) caused by testing errors.

Sample size was estimated using G*Power 3.1 based on the primary hypothesis that chrononutrition indicators would show small-to-moderate associations with body-composition and metabolic outcomes. Using Pearson correlation analysis (α = 0.05, power = 80%, effect size r = 0.25), the minimum required sample size was 128 participants. The subsequent multivariable regression analyses were therefore considered hypothesis-driven extension analyses to further examine the independence and relative contribution of the observed associations. To account for incomplete questionnaires and invalid physiological measurements, 174 participants were recruited. Among them, 133 participants provided valid data for both DXA and RMR assessments (overall attrition for this subsample: 23.6%, *n* = 41; 18 due to incomplete questionnaires, 15 due to invalid test data, and 8 due to voluntary withdrawal). Because different questionnaire-derived chrononutrition indicators required different levels of response completeness, the valid sample size varied across analyses and is reported in the corresponding Results subsections and table footnotes. No significant baseline differences were found between participants included in the DXA + RMR subsample and those excluded from that subsample (all *p* > 0.05).

### 2.2. Ethical Approval

This study was conducted in accordance with the Declaration of Helsinki and was approved by the Ethics Committee of Beijing Sport University (Approval No.: 2024310H). Written informed consent was obtained from all participants prior to the study. All participants were informed of the purpose, procedures, potential risks and benefits of the study, and had the right to withdraw from the study at any time without any adverse consequences.

### 2.3. Data Collection

#### 2.3.1. Questionnaire Survey

Chrononutrition behaviors were assessed using the Chinese version of the Chrononutrition Profile Questionnaire (CP-Q) [22,23], which was translated and culturally adapted following Brislin’s model.(See Appendix A for detailed information on the original source, Chinese adaptation, item summary, and variable derivation.) The questionnaire has demonstrated good test–retest reliability (ICC ≥ 0.75 for 7 of 17 items) and acceptable criterion validity (Pearson r = 0.441–0.922 with sleep and dietary recall measures, *p* < 0.05). The Chinese version used in the present study included 17 items across 4 core dimensions: meal timing, weekday eating window, meal regularity, and energy distribution across meals. The questionnaire was completed by participants via a self-reported online form, with a completion time of approximately 10–15 min. In our sample, internal consistency was good (Cronbach’s α = 0.80).

To control for recall and reporting bias, the following quality control measures were adopted: (1) setting 3 logical check questions (e.g., consistency between sleep time and first meal time) to screen out invalid questionnaires; (2) excluding questionnaires completed in <5 min or >30 min; (3) conducting a telephone recheck for 10% of the questionnaires to verify the consistency of key indicators (ICC ≥ 0.80).

In addition to the CP-Q, demographic and lifestyle information was collected using a supplementary questionnaire, including age, sex, sleep duration, smoking, and alcohol consumption. Physical activity was assessed using the International Physical Activity Questionnaire-Short Form (IPAQ-SF).

The main chrononutrition indicators used in this study were defined as follows. (1) Weekday eating window: The time interval between the first eating event and the last eating event on weekdays (excluding water). Given the fixed training schedule, weekday data were used as the primary indicator to reflect the regular dietary pattern of sports-majoring students. (2) Night eating: Consumption of calorie-containing food from 1 h after sleep onset until the next wake-up. For cross-group comparison, 22:00 was used as a unified reference time point (Supplementary Table S6). (3) Ideal meal/sleep time: The subjective ideal meal and sleep schedule self-reported by participants, without a unified recommended standard. (4) Sleep midpoint time: Calculated as [sleep onset time + (sleep duration/2)], representing the midpoint of the individual’s sleep period in a day. (5) Eating midpoint time: Calculated as [first meal start time + (weekly weighted weekday eating window/2)]. (6) Chronotype discrepancy: Calculated as the signed difference between the actual measured value and the self-reported ideal value for four indicators (sleep duration, sleep midpoint time, eating midpoint time, and weekday eating window). Positive and negative values indicate the direction of deviation from the self-reported ideal schedule. For descriptive presentation, these discrepancy indicators are reported in their original time units (hours). (7) Regular meal days: A single day on which an individual eats the three main meals (breakfast, lunch, dinner) at fixed times (with a tolerance of ±30 min for each meal).

In addition, two derived indicators were calculated in the present study. Chrononutrition disturbance score: Calculated via the equal-weight summation method based on the Z-score standardized absolute deviation values of four indicators (sleep midpoint, eating midpoint, weekday eating window, and sleep duration), with higher scores indicating more severe chrononutrition disturbance. Specifically, each indicator accounted for 25% of the total score. Critical score cut-offs were defined using the percentile method: 0–10 points (40.8%, mild disturbance), 10–20 points (22.5%, moderate disturbance), 20–30 points (14.8%, severe disturbance), and ≥30 points (21.8%, extreme disturbance).

#### 2.3.2. Body Composition Testing

Dual-energy X-ray absorptiometry (DXA) was performed to accurately assess body composition indicators using an iDXA bone densitometer (GE Lunar, Madison, WI, USA). Prior to testing, strict equipment calibration procedures were implemented in accordance with the manufacturer’s guidelines: daily calibration was completed using the proprietary calibration module, including sequential scanning positioning calibration, density calibration, and dose calibration. This ensured the measurement error of bone mineral density was ≤±2% and that of body composition percentage was ≤±1%. Subject measurements were only initiated after successful calibration, with all calibration parameters recorded for documentation.

Stringent pre-measurement preparations were required for all participants: they were instructed to fast, empty their bladders, and remove all metallic items to avoid interference with scanning results. All subjects underwent a full-body DXA scan (scanning range from skull to heel) in a supine position at a scanning speed of 10 mm/s. All scans were conducted between 8:00 and 12:00 a.m. to eliminate potential confounding effects of testing time on body composition outcomes. Scanning operations were performed by professionally trained technicians. The instrument automatically analyzed and outputted the absolute mass (kg) and relative percentage (%) of total body muscle mass, fat mass, and fat-free soft tissue (mainly including bone mineral content, water, and visceral non-adipose/non-muscle tissue). The detection precision of the DXA system was verified by repeated scanning of 10 healthy volunteers, with the coefficient of variation (CV) for muscle mass percentage and fat mass percentage being ≤1.0% and ≤0.8%, respectively. No extreme values of body composition indicators were excluded in this study, as all measured values fell within the normal physiological range. Two core body composition indicators were focused on in the subsequent analyses: muscle mass percentage and fat mass percentage, with limb muscle mass (kg) and visceral fat area (cm^2^) as secondary indicators for stratified analysis (results in Supplementary Material Table S7).

#### 2.3.3. Resting Metabolic Rate Testing

Indirect calorimetry was used to accurately measure all energy metabolism indicators with a portable gas metabolism analyzer (CORTEX Metalyzer 3B, Cortex Biophysik GmbH, Leipzig, Germany). The testing environment was controlled under standard laboratory conditions: temperature maintained at 20~24 °C, relative humidity at 40~60%, no obvious air flow, ambient noise ≤50 dB, soft light without direct glare, and the laboratory was ventilated in advance to ensure stable air composition. A quiet and comfortable testing condition was provided for the subjects to avoid environmental factors interfering with respiratory metabolism data.

Before the test, the equipment was calibrated according to the standard process to ensure measurement accuracy: Gas calibration: Zero calibration and span calibration of the gas sensor were performed using standard gases provided by the manufacturer (oxygen concentration 16.00%, carbon dioxide concentration 0.04%), ensuring that the error of oxygen concentration measurement was ≤±0.1% and the error of carbon dioxide concentration measurement was ≤±0.01%; Flow calibration: A 3 L standard volume syringe was used to calibrate the flow sensor at three flow gradients (30 L/min, 60 L/min, 90 L/min) to verify the accuracy of flow measurement with an error ≤±2%; Heart rate calibration: The heart rate monitoring module of the equipment was connected to a standard heart rate simulator to verify the synchronization and accuracy of heart rate data collection, with the error controlled within ±1 beat per minute. All calibration processes were recorded and archived for traceability.

RMR measurement process: The subjects fasted for at least 10 h the night before the test, avoided strenuous exercise, caffeine, alcohol and tobacco intake within 24 h before the test, and the test was conducted in a quiet and temperature-appropriate laboratory. Participants were placed in a supine position, fully relaxed, and breathed calmly through a mask for 40 min. The first 10 min of data were discarded (adaptation phase), and the average value of the last 30 min of stable data was used for RMR calculation. Stable state was defined as a coefficient of variation (CV) ≤10% for oxygen consumption (VO_2_) and carbon dioxide production (VCO_2_) over consecutive 5-min intervals, consistent with standard indirect calorimetry protocols. And the RMR (kcal/d) was calculated using the Weir formula. The specific form of the Weir formula is: RMR (kcal/d) = (3.941 × VO_2_ + 1.106 × VCO_2_) × 1440/1000. Body-weight-standardized RMR was calculated as RMR divided by body weight (kcal·kg^−1^·d^−1^). The interval between RMR test and DXA test for all subjects was ≤48 h, and both tests were completed in a fasting state. Each subject was tested once, and professional personnel monitored the entire testing process to promptly investigate abnormal data and ensure the accuracy and reliability of the test results.

#### 2.3.4. Quality Control

Strict quality control measures were adopted during the study implementation: (1) All investigators received unified training to standardize the processes of questionnaire distribution, filling guidance and testing operations, so as to avoid human errors; (2) After questionnaire collection, two researchers reviewed them separately to eliminate incomplete and logically contradictory questionnaires, ensuring the quality of the questionnaires; (3) Testing equipment was calibrated regularly (once a quarter), and the equipment status was checked before testing to ensure the accuracy of test data; (4) Double data entry was performed for research data, followed by logical verification and consistency checks to avoid entry errors.

### 2.4. Statistical Analysis

Statistical analyses were performed using SPSS 26.0 (IBM Corp., Armonk, NY, USA) and R 4.4.2 (R Foundation for Statistical Computing, Vienna, Austria). First, the Shapiro–Wilk test was used to verify the normality of continuous variables: normally distributed variables were presented as mean ± standard deviation (SD), and non-normally distributed variables were presented as median (interquartile range, IQR); categorical variables were presented as number (percentage). Between-sex and between-major (sports/non-sports) comparisons were conducted using independent-samples *t* tests (normally distributed continuous variables), Mann–Whitney U tests (non-normally distributed continuous variables) or chi-square tests (categorical variables), according to variable type and distribution. For weekday–weekend comparisons, repeated-measures analyses were performed to test within-subject differences between weekdays and weekends and their interaction with academic major.

Pearson correlation analysis (normally distributed variables) or Spearman correlation analysis (non-normally distributed variables) were used as exploratory analyses to screen unadjusted associations between chrononutrition variables and body-composition or metabolic indicators. FDR correction was applied to exploratory correlation analyses to reduce the risk of Type I error. Multiple linear regression models were then used as the primary analytic approach, with all chrononutrition variables included in the model by the forced entry method. Semi-partial correlation coefficients (sr^2^) were reported to quantify the independent explanatory power of each chrononutrition variable for the dependent variable. Variance inflation factor (VIF) was used for collinearity diagnosis, and all variables had VIF < 3, indicating no severe collinearity. These models were applied to examine independent associations of chrononutrition variables with BMI, fat mass percentage, muscle mass percentage, absolute resting metabolic rate (RMR), and body-weight-standardized RMR after adjustment for age and sex. Because self-reported physical activity frequency was closely related to academic major and was measured at a relatively crude categorical level, it was not included in the primary models but was examined in post hoc sensitivity analyses as an additional covariate. Bonferroni correction was applied to the primary regression analyses to correct for multiple comparisons.

Post hoc sensitivity analyses were conducted to account for the confounding effect of self-reported physical exercise frequency derived from the IPAQ-SF (low: <3 times/week, moderate: 3–5 times/week, high: ≥6 times/week), which replicated the multiple linear regression models for all outcomes with physical exercise frequency as an additional covariate alongside age and sex. Detailed results are in the Supplementary Materials (Table S1); no substantial changes in the direction or statistical significance of key associations were observed, confirming the robustness of primary findings.

Because different questionnaire-derived indicators required different levels of response completeness, the valid sample size varied across analyses. Missing data were handled using complete case analysis for primary analyses. Across all variables included in the analyses, missingness was low (all <15%; for example, BMI: 2.3%, weekday eating window: 4.1%, and muscle mass percentage: 3.5%). To further assess the robustness of the findings, multiple imputation with five imputed datasets was performed as a sensitivity analysis (Supplementary Table S5). Because absolute RMR is strongly determined by body size and body composition, especially fat-free mass, the findings for absolute RMR were interpreted cautiously and were further discussed in light of body-composition dependence.

## 3. Results

Sample sizes varied across analyses according to data availability. Specifically, different chrononutrition indicators required different levels of questionnaire completeness: 169 participants provided valid data for frequency-related behaviors (breakfast consumption, post-meal snacks, and night eating), 150 provided valid weekday eating-window data, 145 had paired valid data for weekday–weekend comparisons, and 142 had sufficient information to calculate chrononutrition disturbance score and discrepancy indicators. Body composition and resting metabolic rate analyses were based on the valid subsample of 133 participants who completed both DXA and RMR assessments. Therefore, the corresponding valid sample size is reported at the beginning of each subsection and in the relevant table footnotes.

### 3.1. General Characteristics of Participants

A total of 174 college students aged 18–24 years were enrolled, including 131 sports-majoring students and 43 non-sports-majoring students, as well as 64 males and 110 females. The overall mean age was 20.5 ± 1.8 years, and the overall mean BMI was 23.3 ± 4.5 kg/m^2^. Males had significantly higher BMI and physical exercise frequency than females (both *p* < 0.001), whereas no significant sex differences were observed in age or sleep duration (all *p* > 0.05). Sports-majoring students had significantly higher physical exercise frequency than non-sports-majoring students (*p* < 0.001), whereas no significant differences were found between the two major groups in age, BMI, or sleep duration (all *p* > 0.05). The general characteristics of the participants by sex and major are shown in Table 1. Sleep-related timing variables are shown in Supplementary Figure S1; no significant between-major differences were observed.

### 3.2. Chrononutrition Characteristics of the Study Participants

Valid data for weekday eating window were available for 150 participants, whereas 169 participants were included in the analyses of breakfast consumption, post-meal snacks, and night eating. The distribution of the most abundant meal and regular meal days was analyzed in the full enrolled sample (n = 174), and 142 participants had sufficient information to derive chrononutrition disturbance score and discrepancy indicators. As summarized in Table 2, the mean weekday eating window was 10.4 ± 3.0 h, with most participants clustered in the 9–12 h range. Breakfast consumption was generally infrequent, with only 14.8% of participants reporting daily breakfast intake. Lunch was the most abundant meal for most students (71.0%), whereas dinner was more often reported as the most abundant meal among sports-majoring students than among non-sports-majoring students (28.5% vs. 15.2%, *p* < 0.05). The weekday eating window presented in Table 2 was based on 150 participants with general chrononutrition data, whereas the weekday–weekend comparison in Supplementary Table S8 adopted a paired subsample of 145 participants.

### 3.3. Chrononutrition Disturbance Score and Discrepancy Indicators Among College Students

Valid data for chrononutrition disturbance score and discrepancy analyses were available for 142 participants. The mean chrononutrition disturbance score was 17.1 ± 14.1 points, and no significant difference was observed between sports-majoring and non-sports-majoring students (*p* > 0.05). The distributions of chrononutrition disturbance score and discrepancy indicators are summarized in Table 2 and Supplementary Figure S3.

### 3.4. Chrononutrition Patterns by Sex and Academic Major

This subsection was based on participants with complete chrononutrition pattern data; the sample sizes in Table 3 referred to the valid subsample for timing and energy proportion analyses. Significant sex differences were observed in chrononutrition behaviors (all *p* < 0.05). Compared with females, males had later average breakfast and dinner times, a longer weekday eating window, fewer regular meal days per week, a lower breakfast energy proportion, and a higher dinner energy proportion. No significant sex difference was observed in average lunch time (*p* > 0.05).

Significant major differences were also observed for selected chrononutrition indicators. Compared with non-sports-majoring students, sports-majoring students had a later average dinner time, a longer weekday eating window, a lower breakfast energy proportion, and a higher dinner energy proportion (all *p* < 0.05), whereas no significant between-major differences were found in average breakfast time or regular meal days per week. Detailed distributions of meal timing and meal energy proportion are presented in Supplementary Figure S4.

### 3.5. Chrononutrition Patterns on Weekdays vs. Weekends

Valid data for weekday/weekend comparison were available for 145 participants. Compared with weekdays, weekends were characterized by a longer eating window, later breakfast timing, and fewer regular meal days (all *p* < 0.001), whereas dinner time did not differ significantly (*p* > 0.05). These weekday–weekend differences were consistent across academic major groups (all interaction *p* > 0.05). This weekday–weekend analysis used a paired valid subsample (n = 145), which differed from the general chrononutrition sample in Table 2. Detailed chronological indicators between weekdays and weekends are summarized in Supplementary Table S8.

### 3.6. Body Composition, Resting Metabolic Rate (RMR) and Body-Weight-Standardized RMR

A total of 133 participants completed body-composition assessment and RMR testing, including 93 sports-majoring students and 40 non-sports-majoring students, as well as 50 males and 83 females. Significant sex and major differences were observed in muscle mass percentage, fat mass percentage, limb muscle mass, visceral fat area, RMR, and body-weight-standardized RMR (all *p* < 0.05; Table 4). Overall, males and sports-majoring students showed higher muscle mass percentage, limb muscle mass, visceral fat area, RMR, and standardized RMR, and lower fat mass percentage than females and non-sports-majoring students, respectively. Detailed subgroup comparisons of limb muscle mass and visceral fat area are provided in Supplementary Table S7.

### 3.7. Correlation Analysis of Chrononutrition with Body Composition and Metabolic Indicators

Correlation analyses were conducted in the valid sample with complete body composition and RMR data (n = 133). After FDR correction, the observed associations were generally small to modest in magnitude. Night eating frequency was positively associated with BMI, whereas weekday eating window was negatively associated with fat mass percentage. Sleep-duration discrepancy was negatively associated with muscle mass percentage and absolute RMR, while sleep-midpoint discrepancy was positively associated with muscle mass percentage. Overall, chrononutrition indicators showed more consistent associations with body composition than with absolute or standardized RMR. Detailed correlation coefficients are presented in Supplementary Figure S5. In addition, night eating was further defined as food intake within 1 h after sleep onset for sensitivity verification, with detailed correlation results presented in Supplementary Table S6.

### 3.8. Multiple Linear Regression Analysis of the Associations of Chrononutrition with Body Composition and Metabolic Indicators

**Figure 1:**
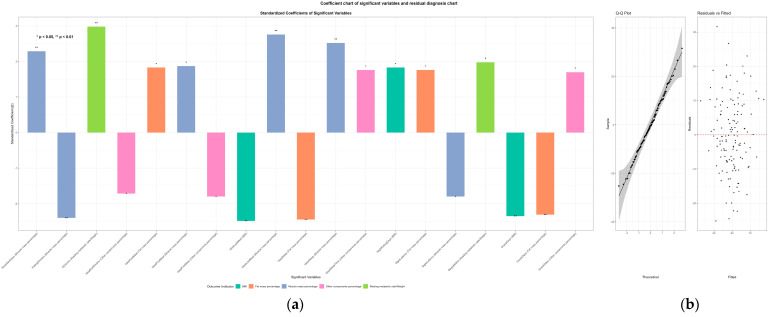
Standardized coefficient chart of significant chrononutrition variables and residual diagnosis of multiple linear regression models. (**a**) Bar chart of standardized coefficients for significant chrononutrition variables in multiple linear regression models (adjusted for age/sex, Bonferroni-corrected *p* < 0.05) for core dependent variables; positive/negative bars indicate the direction of association; (**b**) Residual diagnosis plots (Q-Q plot + Residuals vs. Fitted Values) for the regression models, confirming the normality and homogeneity of residuals (meeting linear regression assumptions). Only core dependent variables (excluding exploratory other components percentage) are presented.

Multiple linear regression analyses were conducted in the valid sample with complete body composition and RMR data (n = 133), with all models adjusted for age and sex and *p*-values corrected for multiple comparisons. As shown in Figure 1 and Supplementary Table S9, chrononutrition variables were independently associated primarily with body composition outcomes rather than with absolute RMR. In particular, breakfast frequency was positively associated with muscle mass percentage, whereas night eating frequency was positively associated with BMI. Several timing- and discrepancy-related indicators were additionally associated with muscle mass percentage, fat mass percentage, BMI, and body-weight-standardized RMR. No significant major-specific interaction effects were observed. Detailed interaction analyses are presented in Supplementary Table S4, and exploratory results for other components percentage are shown in Supplementary Table S3. The main associations remained robust after further adjusting for physical activity frequency, sex stratification, and multiple imputation (Supplementary Tables S1, S4 and S5).

## 4. Discussion

This cross-sectional study investigated the chrononutrition patterns of predominantly sports-majoring Chinese college students and their associations with body composition and resting metabolic rate (RMR). The key findings revealed that chrononutrition behaviors were more consistently correlated with body composition indicators than with absolute RMR in this population; frequent night eating was positively associated with BMI, and regular breakfast consumption was positively associated with muscle mass percentage. Additionally, significant sex differences were observed in chrononutrition patterns, and no notable major-specific interaction effects were found, indicating the core associations were consistent across sports and non-sports majors (with the overall results mainly driven by the sports-majoring population). These findings supplement the chrononutrition research data for Chinese college students with high physical activity levels and provide a targeted theoretical basis for formulating metabolic health promotion strategies for this unique group and suggest that chrononutrition may be more relevant to body composition than to absolute resting metabolism. A key strength of this study is its focus on sports-majoring college students—a physically active population with distinct metabolic and body compositional phenotypes. Unlike non-sports students, sports majors exhibit unique chrononutrition patterns driven by training-related energy needs, including longer weekday eating windows and higher evening energy intake. These behaviors are adaptive, not pathological. Therefore, our findings cannot be generalized to the general student population, highlighting the importance of population-specific chrononutrition research.

A notable feature of the present study was the high prevalence of breakfast irregularity and the tendency for energy intake to be shifted away from the morning. This pattern is metabolically relevant because recent chrononutrition research increasingly supports the view that earlier, more regular, and better-aligned eating schedules are more compatible with endogenous circadian rhythms [3,4]. The temporal eating patterns observed in our sample are also broadly consistent with previous findings in Chinese college students, among whom irregular meal timing and social jet lag appear to be common [21]. Thus, the breakfast irregularity and delayed eating pattern observed in our sample may represent a broader disruption of circadian eating organization rather than an isolated dietary habit.

However, the muscle mass percentage in this study (64.6 ± 12.7%) is higher than that in ordinary Chinese college students (about 55–60%), which is due to the sports-related majors of the participants, reflecting the influence of physical activity on body composition. The longer weekday eating window of the subjects may be related to the higher daily energy demand of sports college students and multiple energy supplements after exercise. Therefore, the extrapolation of this study’s conclusions to ordinary college students needs to be comprehensively judged in combination with physical activity level.

The positive association between night eating and BMI observed in our study is broadly consistent with current evidence. In a recent longitudinal study of 48,150 Chinese adults, frequent skipping of breakfast and/or night eating was associated with faster increases in body weight and waist circumference over four years, even after adjustment for total energy intake and diet quality [24]. Although the present study was cross-sectional and cannot establish causality, the consistency in direction strengthens the plausibility of our findings. One likely explanation is that food intake later in the day occurs during a circadian phase characterized by reduced glucose tolerance, lower insulin sensitivity, and less favorable postprandial metabolic handling, which may promote positive energy balance and adiposity over time [3,4]. In addition, night eating often clusters with other unfavorable behaviors, such as delayed sleep timing and irregular daily routines, which may further amplify obesity risk [25].

Another notable finding was that the number of breakfast days per week was positively associated with muscle mass percentage. Although most chrononutrition studies have focused on obesity or cardiometabolic risk, this result is biologically plausible. Regular breakfast consumption may help distribute energy and protein intake more evenly across the day, reduce prolonged morning fasting, and support synchronization of peripheral metabolic clocks, including those in skeletal muscle [3,4]. Supporting this interpretation, Wu et al. reported in young adults that earlier and more regular temporal eating patterns were associated with more favorable body-composition indicators [26]. Mao et al. found that chrononutrition behaviors, including eating-window characteristics and timing of intake, were associated with muscle mass and function in older adults [27]. Because our study did not directly measure total protein intake or protein distribution, this interpretation should remain cautious and mechanistic rather than definitive.

The associations involving weekday eating window and body composition deserve cautious interpretation. In our study, a longer weekly average weekday eating window was negatively associated with muscle mass percentage in the adjusted model, while the simple correlation with fat mass percentage also indicated a significant relationship. However, evidence from the literature is not entirely consistent. Wu et al. observed that earlier and more regular temporal eating patterns were linked to more favorable body composition in young adults [26], whereas Dote-Montero et al. reported that meal timing was not clearly related to body composition in young adults, although a longer weekday eating window and a shorter interval from midsleep to first food intake were associated with better cardiometabolic health in men [6]. Moreover, a recent meta-analysis suggested that early time-restricted eating can reduce body weight and fat mass while not clearly compromising fat-free mass [28]. Taken together, these findings imply that the meaning of “weekday eating window” may differ substantially between structured intervention studies and free-living observational settings. In our cohort, a longer weekday eating window may reflect a more irregular intake pattern across the day rather than a deliberate early time-restricted eating strategy. In our sports-majoring cohort, a longer weekday eating window may partly reflect training-related meal extension or post-exercise intake, but this interpretation should be made cautiously because we did not directly assess total energy intake, meal composition, or protein distribution. It is also possible that a longer weekday eating window captured a more dispersed or less regular eating pattern across the day. Therefore, the observed negative association with muscle mass percentage should not be overinterpreted mechanistically and may reflect the combined influence of training schedules, eating distribution, and unmeasured lifestyle factors. Therefore, this finding should not be interpreted as direct evidence against time-restricted eating, but rather as an indication that under habitual college-life conditions, a more extended and potentially disorganized eating span may be linked to less favorable body-composition characteristics.

We also observed that discrepancy-related indicators, especially sleep midpoint discrepancy and sleep duration discrepancy, were associated with body composition and standardized RMR. This is meaningful because chrononutrition does not act in isolation; rather, it is embedded within a broader circadian behavioral system that includes sleep timing, wake timing, light exposure, and daily schedules [3,4]. This interpretation is supported by a systematic review showing that evening chronotype is associated with obesity risk and less favorable weight-control outcomes [25]. Accordingly, our findings suggest that eating-related rhythms and sleep-related misalignment may jointly shape metabolic phenotypes in college students. From a practical perspective, improving eating timing without addressing irregular sleep–wake patterns may produce only limited benefits.

The RMR-related findings require restrained interpretation. In the present study, chrononutrition variables did not independently predict absolute RMR after adjustment for age and sex, whereas standardized RMR remained associated with chrononutrition disturbance score and sleep midpoint discrepancy. This pattern is reasonable because absolute RMR is strongly determined by body size and body composition, especially fat-free mass, and may therefore be less sensitive to the independent contribution of meal timing in a relatively young and generally healthy sample. In contrast, standardized RMR may better reflect relative metabolic efficiency after accounting, at least partly, for interindividual differences in body weight. Therefore, our data suggest that chrononutrition may be more closely related to relative metabolic phenotype and body-composition patterning than to absolute resting energy expenditure itself. This interpretation is aligned with the broader literature, where evidence linking meal timing to adiposity is generally more consistent than evidence for large direct effects on absolute resting metabolism [3,4,28].

This study has several strengths. First, body composition was assessed using DXA and resting metabolism was measured by indirect calorimetry, which improved phenotypic precision compared with studies relying only on BMI or predictive equations. Second, we assessed multiple dimensions of chrononutrition simultaneously, including meal timing, weekday eating window, breakfast frequency, night eating, and discrepancy-related indicators, allowing a relatively comprehensive evaluation of temporal eating behaviors in college students. Third, this study contributes data from a Chinese young-adult population, which remains underrepresented in the chrononutrition literature.

Several limitations should also be acknowledged. First, this study is a cross-sectional design, which cannot determine the causal relationship between chrononutrition behaviors and outcome indicators (e.g., it cannot be judged whether night eating leads to higher BMI or subjects with higher BMI are more likely to form night eating habits), and prospective cohort studies are needed for verification in the future. Second, chrononutrition behaviors were self-reported and may be affected by recall bias or reporting bias. Third, although sensitivity analyses additionally adjusted for self-reported physical activity frequency, residual confounding related to training load, exercise type, or overall lifestyle cannot be excluded. Fourth, the participants were recruited from a relatively homogeneous university population, which may limit generalizability to other age groups or non-student populations. In addition, sports-majoring participants were not further classified by specific sport type (e.g., endurance, strength/power, or combined sports), which may have introduced heterogeneity within the sports-majoring group. Finally, some associations in the present study were modest and not always directionally consistent across correlation and regression analyses; therefore, the findings should be interpreted as exploratory rather than definitive. In addition, although absolute RMR was analyzed as an outcome, the primary regression models did not additionally adjust for fat-free mass or other body-composition components, which are major determinants of resting metabolism; therefore, the null findings for absolute RMR should be interpreted with caution.

Taken together, the present findings suggest that breakfast frequency and night eating may be relevant chrononutrition-related correlates of metabolic health in college students. However, because this study was cross-sectional, these observations should be interpreted cautiously and require confirmation in prospective and intervention studies.

## 5. Conclusions

In conclusion, among this predominantly sports-majoring sample of Chinese college students, chrononutrition behaviors were more consistently associated with body composition than with absolute resting metabolic rate. Frequent night eating was positively associated with BMI, whereas regular breakfast consumption was positively associated with muscle mass percentage. Significant sex differences in chrononutrition patterns were also observed. However, given the cross-sectional design and the sports-university setting, causal inference and broader generalizability remain limited. Future longitudinal and interventional studies are needed to further clarify the relationships between chrononutrition and metabolic health.

## Figures and Tables

**Table 1:** General characteristics of participants by sex and major.

Variables	Total (n = 174)	Male (n = 64)	Female (n = 110)	Sports-Majoring Male (n = 48)	Sports-Majoring Female (n = 83)	Non-Sports-Majoring Male (n = 16)	Non-Sports-Majoring Female (n = 27)	*p*-Value (Sex)	*p*-Value (Major)
Age (years)	20.5 ± 1.8	20.7 ± 1.7	20.3 ± 1.9	20.8 ± 1.6	20.5 ± 1.7	20.3 ± 1.8	20.4 ± 2.0	0.210	0.374
BMI (kg/m^2^)	23.3 ± 4.5	24.8 ± 4.2	22.4 ± 4.4	25.1 ± 4.0	22.6 ± 4.3	23.9 ± 4.5	22.1 ± 4.7	<0.001	0.450
Physical exercise frequency (times/week)	5.2 ± 2.1	5.8 ± 2.0	4.8 ± 2.0	6.2 ± 1.8	5.2 ± 1.9	4.1 ± 1.6	2.8 ± 1.3	<0.001	<0.001
Sleep duration (h/day)	9.0 (7.2, 11.5)	8.8 (7.0, 11.2)	9.1 (7.3, 11.8)	8.7 (6.9, 11.0)	9.0 (7.2, 11.5)	9.1 (7.3, 11.8)	9.3 (7.6, 12.1)	0.286	0.312

Notes: BMI, body mass index. Data are presented as mean ± standard deviation (SD) for normally distributed variables and as median (interquartile range, IQR) for non-normally distributed variables. *p*-value (sex) refers to the comparison between male and female participants; *p*-value (major) refers to the comparison between sports-majoring and non-sports-majoring participants. Between-group comparisons were performed using independent-samples *t* tests, Mann–Whitney U tests, or chi-square tests, as appropriate.

**Table 2:** Summary of chrononutrition characteristics among college students.

Section	Variable	Sample Size (n)	Value/Distribution
A. Frequency-related chrononutrition behaviors	Breakfast consumption	169	Never: 10.1%; 1–3 d/week: 36.7%; 4–6 d/week: 38.5%; daily: 14.8%
	Most abundant meal	174	Breakfast: 2.4%; Lunch: 71.0%; Dinner: 26.0%; Others: 0.6%
	Post-meal snacks	169	Never: 14.8%; 1–3 d/week: 57.4%; 4–7 d/week: 27.8%
	Night eating	169	None: 90.5%; 1 d/week: 4.7%; 2 d/week: 3.6%; ≥3 d/week: 1.2%
B. Temporal pattern summary	Weekday eating window (h)	150	10.4 ± 3.0
	Regular meal days per week (d)	174	4.2 ± 1.5
	Breakfast between 7:00–8:30	174	68.3%
	Dinner later than 19:00	174	41.2%
C. Chrononutrition disturbance and discrepancy indicators	Chrononutrition disturbance score	142	17.1 ± 14.1
	Sleep duration discrepancy (h)	142	−0.5 ± 5.5
	Sleep midpoint discrepancy (h)	142	0.3 ± 3.2
	Eating midpoint discrepancy (h)	142	0.7 ± 3.4
	Weekday eating window discrepancy (h)	142	1.6 ± 5.8

Notes: Data are presented as mean ± SD or percentage, as appropriate. Chrononutrition indicators were derived from the validated Chinese version of the CP-Q. Discrepancy indicators are presented as signed differences (actual minus self-reported ideal value) in hours, whereas the chrononutrition disturbance score was calculated from Z-score standardized absolute deviations.

**Table 3:** Chrononutrition patterns by sex and academic major among college students.

Chrononutrition Indicator	Valid n	Male	Female	*p*-Value (Sex)	Sports-Majoring	Non-Sports- Majoring	*p*-Value (Major)
Average breakfast time	174	7:58 ± 0:42	7:46 ± 0:46	<0.05	7:54 ± 0:45	7:49 ± 0:44	0.482
Average dinner time	174	18:55 ± 0:50	18:41 ± 0:53	<0.05	18:52 ± 0:51	18:38 ± 0:52	<0.05
Weekday eating window (hours)	150	11.5 ± 1.7	10.9 ± 1.8	<0.05	11.2 ± 2.8	8.5 ± 2.5	<0.001
Regular meal days per week (days)	174	3.9 ± 1.6	4.5 ± 1.4	<0.05	4.3 ± 1.5	4.0 ± 1.6	0.267
Breakfast energy proportion (%)	174	17.8 ± 5.5	19.4 ± 5.0	<0.05	18.2 ± 5.4	19.8 ± 5.1	<0.05
Dinner energy proportion (%)	174	37.9 ± 6.9	33.2 ± 6.8	<0.05	37.2 ± 6.9	30.5 ± 6.5	<0.001

Notes: Data are presented as mean ± SD; *p*-value (Sex) refers to the comparison between male and female participants, and *p*-value (Major) refers to the comparison between sports-majoring and non-sports-majoring participants. Between-group comparisons were performed using independent-samples *t* tests. Abbreviation: SD, standard deviation. Sample sizes varied across analyses according to data availability. Valid n for weekday eating window = 150; valid n for all other chrononutrition indicators = 174.

**Table 4:** Body composition, RMR and standardized RMR of participants by sex and academic major.

Variables	Total (n = 133)	Male (n = 50)	Female (n = 83)	Sports-Majoring (n = 93)		Non-Sports-Majoring (n = 40)		*p*-Value (Sex)	*p*-Value (Major)
				Male (n = 34)	Female (n = 59)	Male (n = 16)	Female (n = 24)		
Muscle mass percentage (%)	64.6 ± 12.7	73.1 ± 9.4	59.5 ± 11.8	74.2 ± 9.1	62.5 ± 11.5	70.8 ± 9.8	53.1 ± 12.2	<0.001	<0.001
Fat mass percentage (%)	23.6 ± 9.3	17.2 ± 6.9	27.6 ± 8.3	16.1 ± 6.7	24.6 ± 8.5	19.5 ± 7.2	32.8 ± 8.8	<0.001	<0.001
Limb muscle mass (kg)	26.8 ± 11.5	36.5 ± 8.2	20.7 ± 6.3	37.2 ± 8.0	24.1 ± 6.5	35.0 ± 8.5	17.8 ± 6.1	<0.001	<0.001
Visceral fat area (cm^2^)	85.2 ± 41.3	112.5 ± 38.6	68.3 ± 32.5	108.3 ± 37.2	58.2 ± 31.8	121.4 ± 39.8	80.5 ± 33.2	<0.001	<0.001
Other components percentage (%)	11.8 ± 7.8	9.7 ± 4.3	13.0 ± 9.1	9.5 ± 4.2	12.9 ± 9.0	10.1 ± 4.5	13.4 ± 9.2	<0.05	0.422
RMR (kcal/d)	1982.0 ± 593.4	2522.3 ± 440.4	1646.4 ± 401.3	2586.4 ± 435.2	1798.3 ± 405.6	2386.2 ± 442.8	1502.5 ± 398.7	<0.001	<0.001
Standardized RMR (kcal·kg^−1^·d^−1^)	29.9 ± 7.1	32.7 ± 6.5	28.2 ± 6.9	33.2 ± 6.3	30.1 ± 6.8	31.6 ± 6.6	26.3 ± 6.9	<0.01	<0.001

Notes: Data are presented as mean ± SD; *p*-value (sex) refers to the comparison between male and female participants, and *p*-value (major) refers to the comparison between sports-majoring and non-sports-majoring participants. Between-group comparisons were performed using independent-samples *t* tests. Abbreviations: RMR, resting metabolic rate; standardized RMR, body-weight-standardized resting metabolic rate; SD, standard deviation. This table presents the valid subsample (n = 133) with complete body composition and resting metabolic rate data.

## Data Availability

The original contributions presented in this study are included in the article and its Supplementary Material. The raw data supporting the conclusions of this article will be made available by the corresponding author upon reasonable request, due to privacy and ethical restrictions related to human participant data. Informed consent was obtained from all subjects involved in the study. Written informed consent has been obtained from the patients to publish this paper.

## Supplementary Materials

The following supporting information can be downloaded at: https://www.mdpi.com/article/10.3390/nu18081214/s1; Table S1: Post-hoc Sensitivity Analysis: Multiple Linear Regression of Chrononutrition Behaviors on Body Composition and Metabolic Indicators (Adjusted for Age, Sex, and Self-Reported Physical Exercise Frequency); Table S2: Exploratory Correlation Analysis Results of Chrononutrition Indicators with Other Components Percentage; Table S3: Multiple Linear Regression Results of Chrononutrition Indicators with Other Components Percentage (Adjusted for Age and Sex; Bonferroni-Corrected); Table S4: Sex-Stratified Multiple Linear Regression Results of Chrononutrition Behaviors on Core Body Composition Indicators (Adjusted for Age; Bonferroni-Corrected); Table S5: Multiple Imputation Sensitivity Analysis Results of Chrononutrition on Body Composition; Table S6: Correlation Analysis of Night Eating (Sleep Onset Definition) with Body Composition Indicators; Table S7: Limb Muscle Mass and Visceral Fat Area of Participants by Sex and Major; Table S8: Chrononutrition Indicators on Weekdays vs. Weekends (Mean ± SD); Table S9: Multiple linear regression results of chrononutrition variables on body composition and metabolic indicators in the valid sample with complete body composition and RMR data (n = 133) (adjusted for sex and age)]; Figure S1: Distribution of sleep duration among college students; Figure S2: Distribution of weekday eating window duration and comparison of morning/night latency; Figure S3: Distribution of chrononutrition disturbance score and chronotype discrepancy indicators; Figure S4: Distribution of meal timing and meal energy proportion among college students; Figure S5: Correlation heatmap and significant coefficient bar chart of chrononutrition indicators with body composition and metabolic indicators.

## Abbreviations

The following abbreviations are used in this manuscript:

RMR	Resting Metabolic Rate
DXA	Dual-Energy X-ray Absorptiometry
BMI	Body Mass Index
ICC	Intraclass Correlation Coefficient
FDR	False Discovery Rate
VIF	Variance Inflation Factor
CV	Coefficient of Variation
VO_2_	Oxygen Consumption
VCO_2_	Carbon Dioxide Production
APC	Article Processing Charge
SD	Standard Deviation
IQR	Interquartile Range
h	Hours
min	Minutes
kg	Kilograms
cm	Centimeters
kcal	Kilocalories

## Appendix A. Original Source, Chinese Adaptation, and Item Summary of the Chrononutrition Profile Questionnaire (CP-Q)

Chrononutrition behaviors in the present study were assessed using the Chinese version of the Chrononutrition Profile Questionnaire (CP-Q). The Chinese version was translated and culturally adapted following Brislin’s model and showed acceptable reliability and validity in Chinese college students. The version used in the present study included 17 items across four core dimensions: meal timing, weekday eating window, meal regularity, and energy distribution across meals. This appendix provides the original source, adaptation information, item summary, and the derivation of study variables based on the CP-Q.

### Appendix A.1. Original Questionnaire Source

The original CP-Q was developed and validated by Veronda et al. (2020) [23] to assess six key chrononutrition components: breakfast skipping, largest meal, evening eating, evening latency, night eating, and eating window. The Chinese version was culturally adapted and psychometrically verified by Gong et al. (2024) [22] among Chinese college students, with acceptable test–retest reliability (ICC ≥ 0.75 for most items) and good criterion validity against dietary and sleep assessments.

### Appendix A.2. Translation and Cultural Adaptation

The Chinese CP-Q was translated and adapted using the standard Brislin translation model, including forward translation, expert committee review, back-translation, and pilot testing. Minor wording revisions were made to enhance clarity and cultural suitability for Chinese participants. The original item structure, response options, and computational logic were fully preserved to ensure consistency with the original scale.

### Appendix A.3. Item Summary and Domain Mapping of the CP-Q

**Domain**	**Item Content Summary**	**Response Format**	**Derived Variable in This Study**
Meal timing	Typical wake time, first eating event, lunchtime, last eating event, bedtime on weekdays and weekends	Clock time (24-h format)	Average breakfast time; Average dinner time
Eating window	Time interval between first and last eating event on weekdays and weekends	Hours/minutes	Weekday eating window
Meal regularity	Weekly frequency of breakfast intake; regularity of fixed mealtimes	Days per week (0–7)	Regular meal days per week
Energy distribution	Self-reported largest meal; energy proportion across breakfast, lunch, and dinner	Meal label; percentage (%)	Breakfast energy proportion; Dinner energy proportion
Night eating	Weekly frequency of waking at night to eat	Days per week (0–7)	Night eating frequency
Preference & misalignment	Preferred wake time, eating time, bedtime; perceived ideal schedule	Clock time; time duration	Sleep midpoint; Eating midpoint; Chronotype discrepancy; Chrononutrition disturbance score

### Appendix A.4. Operational Definitions and Variable Derivation Used in the Present Study

① Weekday eating window

Duration between the first eating event and the last eating event on weekdays (excluding water).

② Night eating

Consumption of calorie-containing food from 1 h after sleep onset until the next wake-up. For cross-group comparison, 22:00 was used as a unified reference time point.

③ Sleep midpoint

Sleep midpoint = Sleep onset time + (Sleep duration/2)

④ Eating midpoint

Eating midpoint = First meal start time + (Weekday eating window/2)

⑤ Chronotype discrepancy

Signed differences between the actual measured value and the self-reported ideal value for sleep duration, sleep midpoint, eating midpoint, and weekday eating window. These indicators are presented in hours, and the sign indicates the direction of deviation.

⑥ Chrononutrition disturbance score

Calculated using the equal-weight summation method based on the Z-score standardized absolute deviations of four indicators: sleep midpoint, eating midpoint, weekday eating window, and sleep duration. Each indicator accounted for 25% of the total score; higher scores indicate more severe chrononutrition disturbance.
