# Supplementary material for: Chrononutrition, Body Composition, and Resting Metabolic Rate Among College Students: A Cross-Sectional Study
Source: Nutrients. 2026 Apr 11;18(8):1214. doi: 10.3390/nu18081214 (PMC13119362; doi:10.3390/nu18081214)
Supplement: Supplementary file 1 [file nutrients-18-01214-s001.zip › nutrients-4234770-supplementary.pdf]

Supplementary Materials

Table S1. Post-hoc Sensitivity Analysis: Multiple Linear Regression of Chrononutrition Behaviors on Body Composition and Metabolic Indicators (Adjusted for Age, Sex, and Self-Reported Physical Exercise Frequency)

Dependent Variable	Key Chrononutrition Behavior	Standardized Coefficient (β)	P-value	Significance	Direction of Association	Consistency with Primary Analysis
BMI (kg/m²)	Night eating days/week	0.181	0.049	*	Positive	Consistent
BMI (kg/m²)	Post-meal snack days/week	-0.232	0.007	**	Negative	Consistent
Muscle mass percentage (%)	Breakfast days/week	0.227	<0.01	**	Positive	Consistent
Muscle mass percentage (%)	Weekly average weekday eating window	-0.238	<0.01	**	Negative	Consistent
Fat mass percentage (%)	Post-meal snack days/week	-0.229	<0.01	**	Negative	Consistent
Fat mass percentage (%)	Weekly average night latency	0.174	0.048	*	Positive	Consistent
Standardized RMR ($kcal \cdot kg^{-1} \cdot d^{-1}$)	Overall chrononutrition disturbance score	0.295	0.007	**	Positive	Consistent
Standardized RMR	Sleep midpoint discrepancy	0.196	0.041	*	Positive	Consistent

Dependent Variable	Key Chrononutrition Behavior	Standardized Coefficient (β)	P-value	Significance	Direction of Association	Consistency with Primary Analysis
(kcal·kg^{-1}·d^{-1})						

* This table presents the results of post-hoc sensitivity analyses with physical exercise frequency as an additional covariate, which was self-reported and categorized as low (<3 times/week), moderate (3–5 times/week), and high (≥6 times/week). The analyses were performed to verify the robustness of the primary associations between chrononutrition behaviors and body composition/metabolic indicators. Key associations remained consistent in direction and statistical significance with the primary regression model (adjusted for age and sex only). P<0.05, P<0.01; Only key significant chrononutrition behaviors from the primary analysis are presented.

Table S2. Exploratory Correlation Analysis Results of Chrononutrition Indicators with Other Components Percentage.

Chrononutrition Indicator	Sample Size (n)	Correlation Coefficient (r)	P-value (FDR-adjusted)	Significance	Direction of Association
Weekly average sleep duration	133	0.26	0.003	**	Positive
Sleep duration discrepancy	133	0.30	0.001	**	Positive
Weekday eating window discrepancy	133	0.26	0.005	**	Positive
Eating midpoint discrepancy	133	0.20	0.034	*	Positive
Weekly average weekday eating window	133	-0.20	0.029	*	Negative
Sleep midpoint discrepancy	133	-0.28	0.003	**	Negative

* Other components percentage is defined as the proportion of bone mineral content and non-adipose/non-muscle soft tissue in total body

mass. This analysis is exploratory only and not included in the core conclusions of the study. All results were adjusted for false discovery rate (FDR) to reduce Type I error. *P < 0.05, P < 0.01.

Table S3. Multiple Linear Regression Results of Chrononutrition Indicators with Other Components Percentage (Adjusted for Age and Sex; Bonferroni-Corrected)

Dependent Variable: Other Components Percentage (%)	Adjusted R^2	Chrononutrition Indicator	Standardized Coefficient	Bonferroni-Corrected P-value	Significance	Direction of Association
	0.18	Ideal first meal time	1.80	0.042	*	Negative
		Ideal bedtime	1.76	0.045	*	Positive
		Ideal weekday eating window	1.72	0.048	*	Negative
		Post-meal snack days/week	1.70	0.049	*	Positive

* This table presents exploratory multiple linear regression results for the association between chrononutrition indicators and other components percentage (bone mineral content + non-adipose/non-muscle soft tissue). This variable was not a primary outcome of the study, and the results are provided only as a preliminary reference for subsequent research on chrononutrition and skeletal/non-metabolic body components. All P-values were corrected for multiple comparisons using the Bonferroni method. P < 0.05.

Table S4. Sex-Stratified Multiple Linear Regression Results of Chrononutrition Behaviors on Core Body Composition Indicators (Adjusted for Age; Bonferroni-Corrected)

Dependent Variable	Sex Group	Chrononutrition Indicator	Standardized Coefficient	Bonferroni-Corrected P-value	Significance	Direction of Association
BMI (kg/m²)	Male (n=50)	Night eating days/week	1.85	0.041	*	Positive
BMI (kg/m²)	Male (n=50)	Post-meal snack days/week	-2.38	0.006	**	Negative
BMI (kg/m²)	Female (n=83)	Night eating days/week	1.81	0.045	*	Positive
BMI (kg/m²)	Female (n=83)	Post-meal snack days/week	-2.32	0.007	**	Negative
Muscle mass %	Male (n=50)	Breakfast days/week	2.31	0.005	**	Positive
Muscle mass %	Male (n=50)	Weekly average weekday eating window	-2.42	0.004	**	Negative
Muscle mass %	Female (n=83)	Breakfast days/week	2.27	0.006	**	Positive
Muscle mass %	Female (n=83)	Weekly average weekday eating window	-2.39	0.005	**	Negative
Fat mass %	Male (n=50)	Post-meal snack days/week	-2.29	0.006	**	Negative

Fat mass %	Female (n=83)	Post-meal snack days/week	-2.33	0.007	**	Negative

* This table presents sex-stratified multiple linear regression results to explore the consistency of core associations between chrononutrition behaviors and body composition in male and female participants. The model was adjusted for age, and P-values were Bonferroni-corrected for multiple comparisons. Only key significant chrononutrition behaviors from the primary analysis are presented. *P < 0.05, P < 0.01.

Table S5. Multiple Imputation Sensitivity Analysis Results of Chrononutrition on Body Composition.

**Dependent Variable**	**Chrononutrition Indicator**	**β**	**sr²**	**P-value**	**Significance**	**Direction of Association**	**Consistency with Primary Analysis**
BMI (kg/m²)	Number of night eating days per week	0.178	0.031	0.047	*	Positive	Direction + significance consistent
BMI (kg/m²)	Number of post-meal snack days per week	-0.229	0.052	0.008	**	Negative	Direction + significance consistent
Muscle mass percentage (%)	Number of breakfast days per week	0.225	0.051	0.006	**	Positive	Direction + significance consistent
Muscle mass percentage (%)	Weekly average weekday eating window	-0.235	0.055	0.005	**	Negative	Direction + significance consistent
Muscle mass percentage (%)	Ideal first meal time	0.182	0.033	0.043	*	Positive	Direction + significance consistent

Fat mass percentage (%)	Number of post-meal snack days per week	-0.227	0.051	0.007	**	Negative	Direction + significance consistent
Fat mass percentage (%)	Weekly average night latency	0.172	0.030	0.049	*	Positive	Direction + significance consistent
Fat mass percentage (%)	Ideal sleep time	-0.241	0.058	0.004	**	Negative	Direction + significance consistent

* This table presents model adjusted for age, sex and physical exercise frequency (low/<3 times/week, moderate/3-5 times/week, high/≥6 times/week); 5 imputed datasets used for analysis; n=133 (effective body composition/RMR sample); β=standardized coefficient; sr²=semi-partial correlation coefficient; P-values Bonferroni-corrected; ***P<0.001, *P<0.01, P<0.05

Table S6. Correlation Analysis of Night Eating (Sleep Onset Definition) with Body Composition Indicators

**Chrononutrition Indicator**	**Body Composition Indicator**	**r**	**P-value**	**Significance**	**Direction of Association**
Frequency of night eating (days/week)	BMI (kg/m^2)	0.29	0.001	**	Positive
Frequency of night eating (days/week)	Fat mass percentage (%)	0.21	0.028	*	Positive
Frequency of night eating (days/week)	Muscle mass percentage (%)	-0.19	0.039	*	Negative
Frequency of night eating (days/week)	Limb muscle mass (kg)	-0.18	0.045	*	Negative
Frequency of night eating (days/week)	Visceral fat area (cm^2)	0.26	0.003	**	Positive
Energy intake from night eating (kcal/day)	BMI (kg/m^2)	0.25	0.004	**	Positive

Energy intake from night eating (kcal/day)	Fat mass percentage (%)	0.23	0.012	*	Positive
Energy intake from night eating (kcal/day)	Visceral fat area (cm^2)	0.28	0.002	**	Positive

* This table presents night eating defined as 1 hour after self-reported sleep onset to next morning wake-up (22:00 as unified cross-group reference); n=133 (effective body composition/RMR sample); r=Spearman correlation coefficient; P-values FDR-adjusted; ***P<0.001, *P<0.01, P<0.05.

Table S7. Limb Muscle Mass and Visceral Fat Area of Participants by Sex and Major.

**Variables**	**Total (n=133)**	**Male (n=50)**	**Female (n=83)**	**P-value (Sex)**	**Sports-majoring (n=93)**	**Non-sports-majoring (n=40)**	**P-value (Major)**
Limb Muscle Mass (kg)	26.8±11.5	36.5±8.2	20.7±6.3	***<0.001	28.9±10.8	20.5±7.1	***<0.001
Upper Limb Muscle Mass (kg)	9.7±4.2	13.8±3.1	7.2±2.1	***<0.001	10.5±3.9	7.3±2.3	***<0.001
Lower Limb Muscle Mass (kg)	17.1±7.6	22.7±5.4	13.5±4.2	***<0.001	18.4±7.1	13.2±4.0	***<0.001
Visceral Fat Area (cm^2)	85.2±41.3	112.5±38.6	68.3±32.5	***<0.001	76.5±35.2	102.8±42.6	***<0.001
Skeletal Muscle Mass (kg)	29.5±12.6	40.2±9.1	22.8±7.0	***<0.001	31.8±11.8	22.6±7.2	***<0.001

* This table presents data as mean ± standard deviation (SD); n=133 (effective body composition/RMR sample, 70% sports-majoring: n=93, 30%

non-sports-majoring: n=40); independent-samples t-test for sex/major comparison; Skeletal Muscle Mass = total body skeletal muscle mass (core index for muscle mass percentage calculation); ***P<0.001, *P<0.01, P<0.05

Table S8. Chrononutrition Indicators on Weekdays vs. Weekends (Mean±SD).

Chrononutrition Indicator	Weekdays	Weekends	P-value
Average breakfast time (h:min)	7:38±0:45	8:25±0:51	***<0.001
Average lunch time (h:min)	12:05±0:38	12:18±0:42	0.086
Average dinner time (h:min)	18:48±0:52	18:55±0:55	0.102
Weekday eating window (h)	10.6±2.7	11.8±2.9	***<0.001
Regular meal days (days)	4.9±1.4	2.8±1.3	***<0.001
Breakfast energy proportion (%)	18.8±5.2	17.5±4.8	**<0.01
Dinner energy proportion (%)	34.8±7.0	37.3±6.8	**<0.01
Morning latency (h)[1]	0.8±0.4	1.5±0.6	***<0.001
Night latency (h)[2]	2.1±0.7	1.8±0.6	**<0.01

* n=145 (valid weekday/weekend chrononutrition sample, different from body composition sample due to partial questionnaire missing); paired t-test for weekday/weekend comparison; no significant major×weekday/weekend interaction effects (all P>0.05); [1]Morning latency = interval from waking to the first meal; [2]Night latency = interval from the last meal to sleep onset; ***P<0.001, *P<0.01, P<0.05.

Table S9. Multiple linear regression results of chrononutrition variables on body composition and metabolic indicators in the valid sample with complete body composition and RMR data (n = 133) (adjusted for sex and age).

Dependent Variable	Adjusted R^2	Significant Chrononutrition Variables	β	sr^2	P
RMR	0.46	None	—	—	—
Standardized RMR	0.20	Chrononutrition disturbance score	2.98	0.06	0.008
		Sleep midpoint discrepancy	1.98	0.04	0.042
Muscle mass %	0.41	Ideal last meal time	2.76	0.05	<0.01

		Ideal sleep time	2.52	0.05	<0.01
		Weekday eating window	−2.40	−0.05	<0.01
		Breakfast days/week	2.29	0.05	<0.01
		Ideal first meal time	1.87	0.03	0.045
		Night latency	−1.80	−0.03	0.048
Fat mass %	0.29	Ideal sleep time	−2.45	−0.05	<0.01
		Snack days/week	−2.31	−0.05	<0.01
		Ideal first meal time	1.83	0.03	0.046
		Night latency	1.76	0.03	0.049
BMI	0.20	Ideal last meal time	−2.49	−0.05	<0.01
		Snack days/week	−2.35	−0.05	<0.01
		Night eating days/week	1.83	0.03	0.046

*Multiple linear regression results for the associations of chrononutrition variables with body composition and metabolic indicators in the valid sample with complete body composition and RMR data (n = 133), adjusted for age and sex. All chrononutrition variables were entered simultaneously using the forced-entry method. P-values were Bonferroni-corrected for multiple comparisons. VIF values were < 3 in all models, indicating no severe multicollinearity. Other components percentage was treated as an exploratory outcome and is not included in the core conclusions.

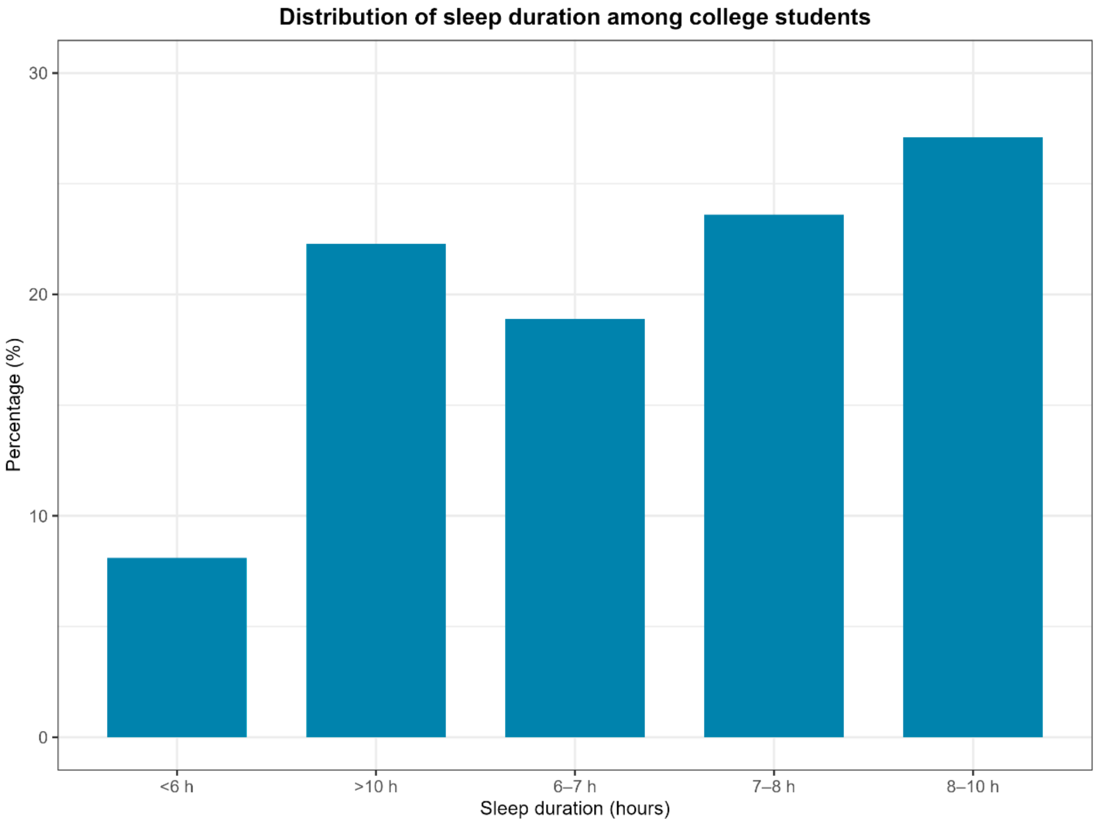

Figure S1. Distribution of sleep duration among college students

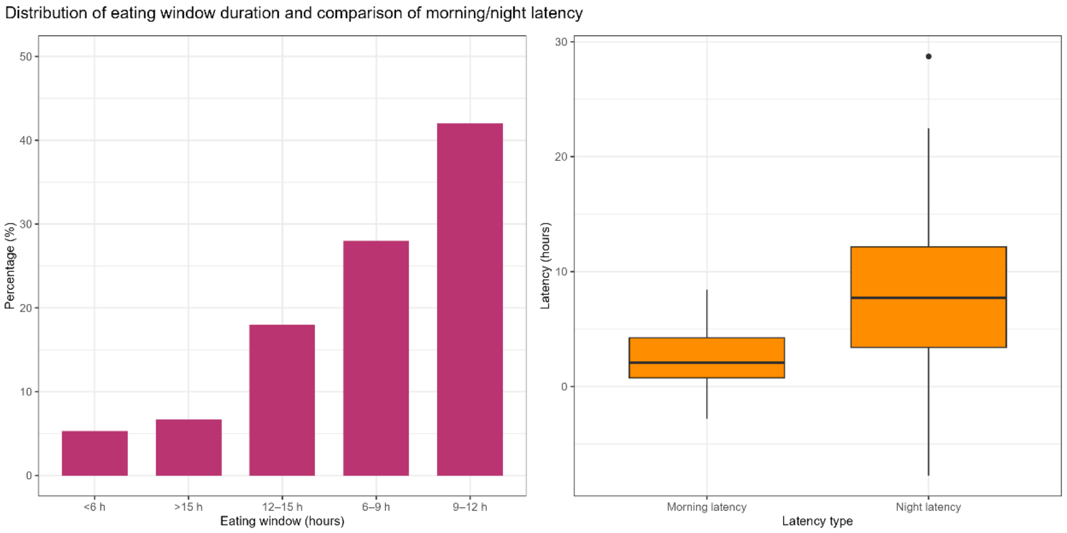

(a) (b)

Figure S2. Distribution of weekday eating window duration and comparison of morning/night latency in the study population: (a) Percentage distribution of weekly average weekday eating window duration (h/day), categorized as <6 h, 6–9 h, 9–12 h, 12–15 h, >15 h; (b) Comparison of weekly average morning latency and night latency (h). Morning latency = interval from waking to the first meal; Night latency = interval from the last meal to sleep onset. Data are presented as mean ± SD; $P<0.01$ for the comparison between morning and night latency (paired t-test).

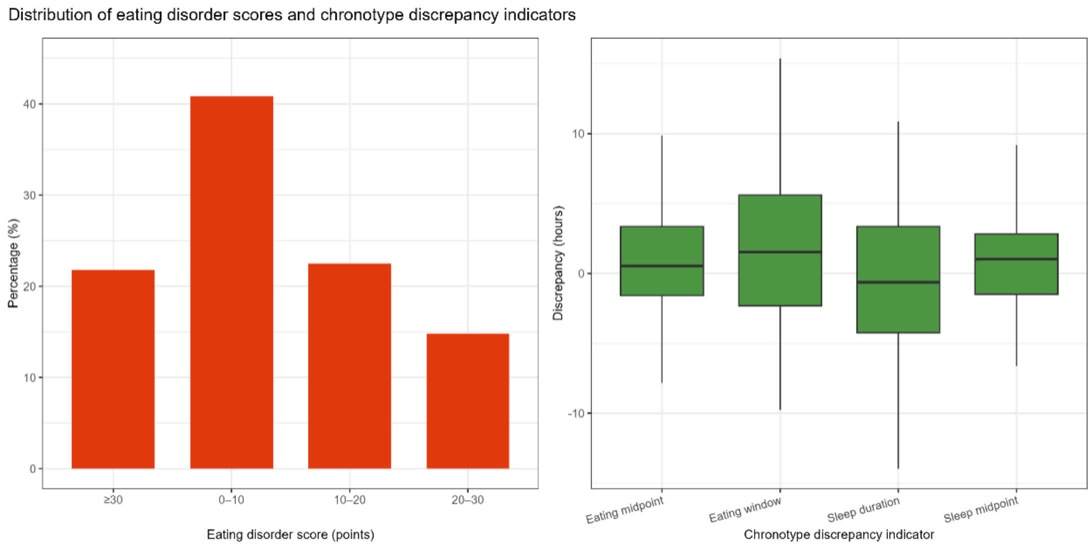

(a) (b)

Figure S3. Distribution of chrononutrition disturbance score and chronotype discrepancy indicators in the study population. (a) Percentage distribution of chrononutrition disturbance score, categorized as 0–10 points (mild disturbance), 10–20 points (moderate disturbance), 20–30 points (severe disturbance), ⩾30 points (extreme disturbance); (b) Distribution of four chronotype discrepancy indicators (h), including sleep duration discrepancy, sleep midpoint discrepancy, eating midpoint discrepancy, weekday eating window discrepancy. Discrepancy = absolute Z-score standardized difference between actual and self-reported ideal values; data are presented as mean ± SD.

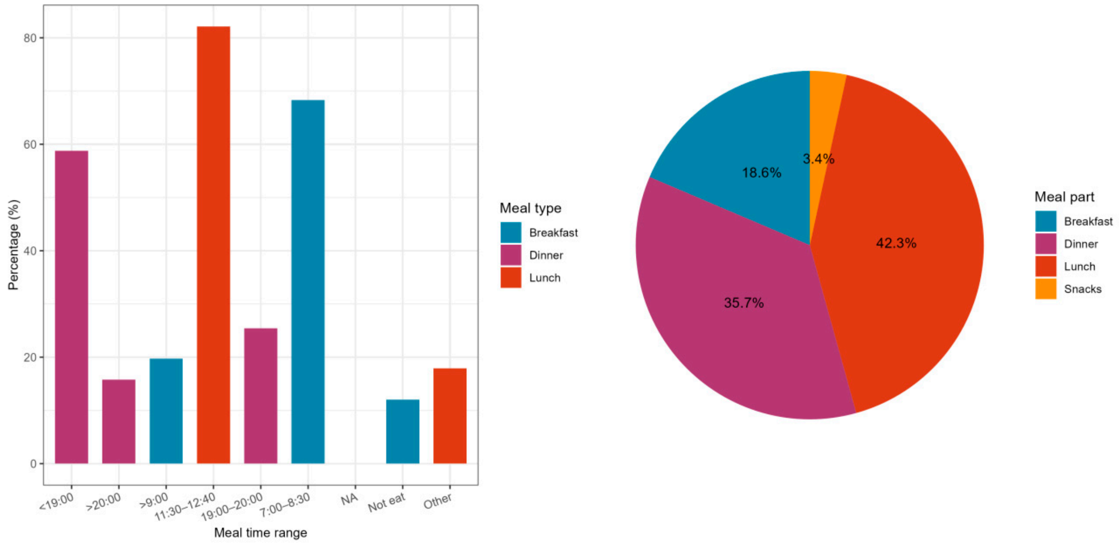

(a) (b)

Figure S4. Distribution of meal timing and meal energy proportion among college students. (a) Percentage distribution of meal time for breakfast, lunch and dinner (categorical time ranges); (b) Energy proportion (%) of breakfast, lunch, dinner and snacks in total daily energy intake. Data are presented as mean ± SD; different lowercase letters indicate significant differences in energy proportion among meal types ($P<0.05$, one-way ANOVA).

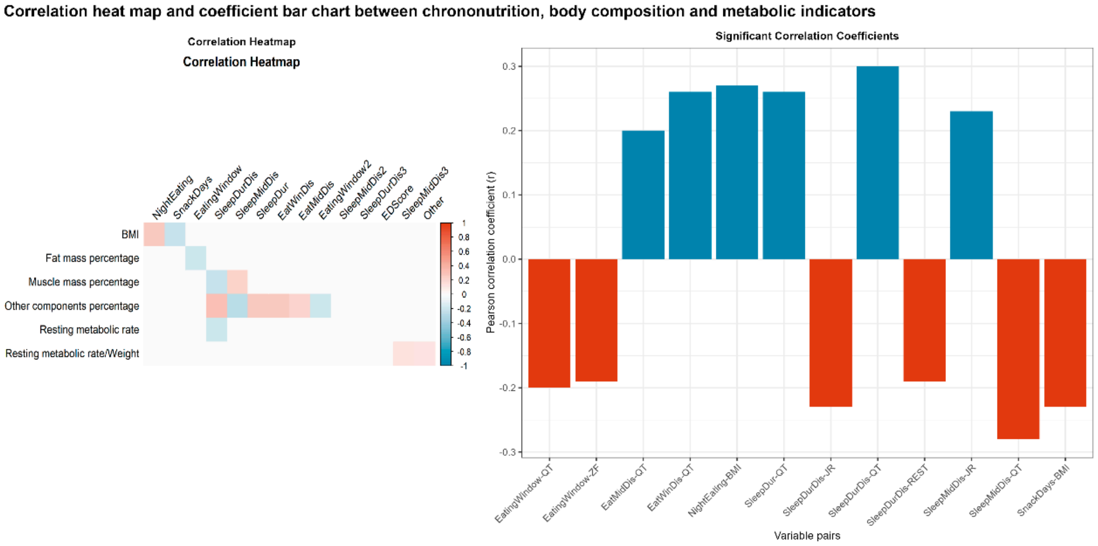

(a) (b)

Figure S5. Correlation heatmap and significant coefficient bar chart of chrononutrition indicators with body composition and metabolic indicators in the valid sample with complete body composition and RMR data (n = 133). (a) Correlation heatmap showing the bivariate correlation between core chrononutrition indicators, body composition and metabolic indicators; red = positive correlation, blue = negative correlation, color depth = correlation strength (|r|); (b) Bar chart of significant correlation coefficients (FDR-adjusted P<0.05) for key variable pairs. All P-values were corrected for false discovery rate (FDR) to reduce Type I error; only core outcomes (excluding exploratory other components percentage) are presented.
